# Ring Fracture of Brazilian Aortic Valve Bioprostheses Using Non-Compliant High-Pressure Transcatheter Balloon, an *Ex Vivo* Test

**DOI:** 10.21470/1678-9741-2021-0272

**Published:** 2022

**Authors:** Andre Lupp Mota, Diego Felipe Gaia, José Honório Palma da Fonseca

**Affiliations:** 1 Division of Cardiac Surgery, Escola Paulista de Medicina, Universidade Federal de São Paulo, São Paulo, São Paulo, Brazil.

**Keywords:** Aortic Valve, Transcatheter Aortic Valve, Cardiovascular Diseases, Bioprosthesis, Balloon Valvuloplasty

## Abstract

**Introduction:**

Aortic valve bioprostheses ring fracture in valve-in-valve procedures has shown low complication rates and presents as an option in the treatment of patients at high risk for conventional surgery, avoiding high transvalvular gradients, which are associated with increased mortality. Some prostheses available in the market cannot be fractured. In an ex vivo test, the possibility of ring fracture of aortic valve bioprostheses produced in Brazil when submitted to radial force application using a high-pressure non-compliant balloon was evaluated.

**Methods:**

One unit of each aortic valve bioprosthesis model, sizes 19 and 21 mm, produced by Brazilian companies (Braile Biomédica, Cardioprótese, and Labcor), was used. In the experiment, a non-compliant high-pressure balloon (Atlas®-Gold), 1 mm larger than the external diameter of the prosthesis, was positioned inside the valve annulus and inflated gradually aiming to fracture the prosthesis. Fracture pressures and photographic and radiological images of the prostheses before and after test were recorded.

**Results:**

All prostheses were fractured. In the models with metal ring, the fracture pressures were between 23 and 25 atm. In the other prostheses, the rupture occurred between 10 and 13 atm. No deformations in the structure were observed, which could potentially damage the aortic root.

**Conclusion:**

All the Brazilian prostheses evaluated were fractured, although the presence of a metal ring in the prosthesis framework increases the pressure required for fracture. The information obtained helps in the planning of valve-in-valve procedures in patients with aortic valve bioprostheses.

**Table:** 

Abbreviations, Acronyms & Symbols	
TAVI	= Transcatheter aortic valve implantation
VIV	= Valve-in-valve

## INTRODUCTION

Transcatheter aortic valve implantation (TAVI) emerged in 2002 as a minimally invasive treatment of native aortic valve stenosis in patients at high surgical risk for conventional open surgery^[1]^. The adaptation of this technique, performing the implantation of a transcatheter prosthesis inside a bioprosthesis, became known as valve-in-valve (VIV) procedure and allows the treatment of patients with bioprosthetic valve dysfunction, commonly observed about 10 years after implantation^[2,3]^.

VIV procedures frequently result in reduced effective orifice area and prosthesis-patient mismatch, especially when implanted in small bioprosthetic valves (sizes 19-21 mm)^[4]^. When the post- TAVI gradient is > 20 mmHg, it is related to increased mortality in one year after the procedure^[5]^. Bioprosthetic ring fracture using a transcatheter non-compliant high-pressure balloon may increase the valve diameter and therefore its effective orifice area, reducing the gradient without related annular or aortic root rupture, coronary occlusion, or need for pacemaker. In a series with 75 cases submitted to this technique, only two patients presented transcatheter prosthesis insufficiency, resolved with the implantation of a new transcatheter valve^[6-8]^.

Due to the great variety of bioprostheses available in the market, with different types of structures, some *ex vivo* tests have evaluated the possibility of ring fracture and the pressure required for it, if it occurs^[9]^. However, these studies did not include prostheses produced in Brazil. The resulting information is of great importance in the preoperative evaluation of VIV procedures, since some brands do not suffer fractures or may require too high pressures.

## METHODS

The experiment was approved by the ethical committee of the Universidade Federal de São Paulo (number 2686290917). According to the Agência Nacional de Vigilância Sanitária (or Anvisa) electronic query system^[10]^, three Brazilian industries currently produce aortic bioprostheses: Braile Biomédica, Cardioprótese, and Labcor. The experiment was based on the original description of the technique^[11]^ and used one model of each bioprosthesis of nominal sizes 19 and 21 mm (external diameter) of each company. Also, Atlas Gold PTA balloons (Bard Peripheral Vascular Inc.) of 20- and 22-mm diameters were used, aiming a diameter 1 mm larger than the external diameter of the prosthesis to which it was intended. The balloon was introduced in the prosthesis to be fractured, and the balloon insufflation line was connected to one of the paths of a three-way stopcock. The other two paths were connected to an insufflator and a 60-ml capacity syringe, both filled with 0.9% saline solution.

With the stopcock path open to the syringe, the balloon was inflated manually until resistance was reached. Then, the stopcock was opened for the inflator line and the inflation proceeded. Prosthetic ring fracture was characterized by an abrupt drop in the pressure recorded on the inflator manometer, often accompanied by a characteristic high-pitched sound. The pressure at the time of fracture was recorded. Photographs and radiographs (superior-inferior projection) were taken to compare the appearance of the structure before and after the maneuver. Finally, the lining of the prosthesis rings was removed for evaluation and photographic documentation.

## RESULTS

In the experiments performed, all the prostheses were fractured at the first attempt, except the 19-mm Braile prosthesis, which required two attempts with a 20-mm balloon, because the first balloon was ruptured around 24 atm. The ring fracture with the second balloon occurred at 25 atm. It was observed that the pressure required for fracture of the prostheses produced by Braile, with metallic rings, was higher than the breaking pressure of the other models (Table 1).

**Table 1:** Pressures (in atm) at which fracture occurred in each bioprosthesis model studied after application of radial force by a high-pressure non-compliant balloon, sizes 20 or 22 mm.

Model	Fracture pressure (atm)
Cardioprótese Premium® 19 (20-mm balloon)	8
Cardioprótese Premium® 21 (22-mm balloon)	12
Labcor Dokimos Plus® 19 (20-mm balloon)	11
Labcor Dokimos Plus® 21 (22-mm balloon)	12
Labcor® TLPB 19 (20-mm balloon)	7
Labcor® TLPB 21 (22-mm balloon)	11
Braile®19 (20-mm balloon)	25
Braile® 21 (22-mm balloon)	23

**Fig. 1:**
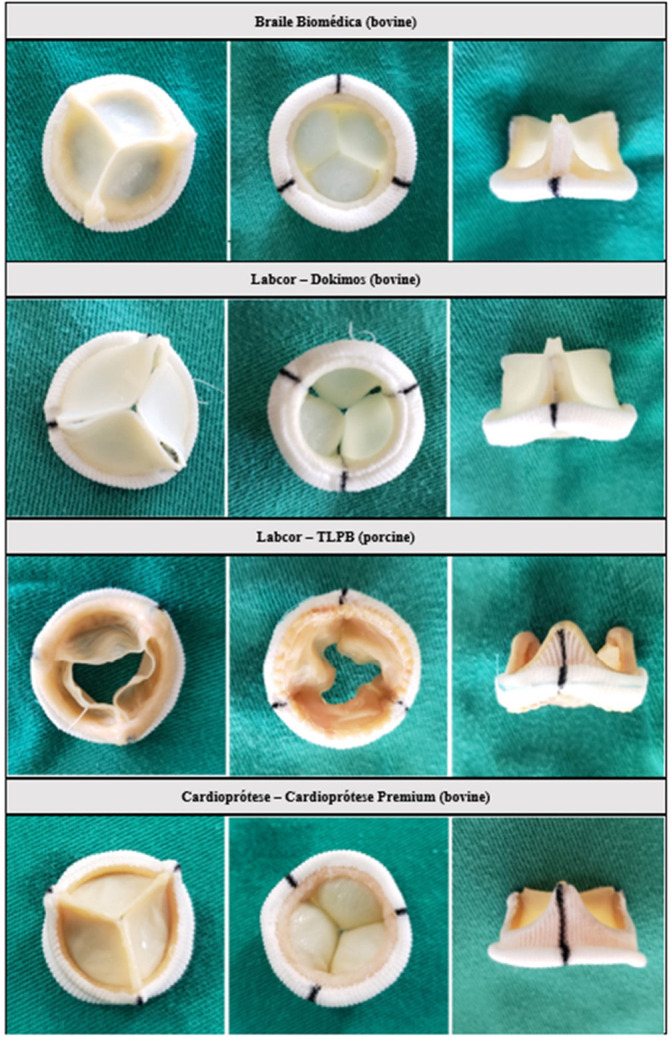
Photographs of the prostheses used in the test in their upper, lower, and lateral aspects.

**Fig. 2:**
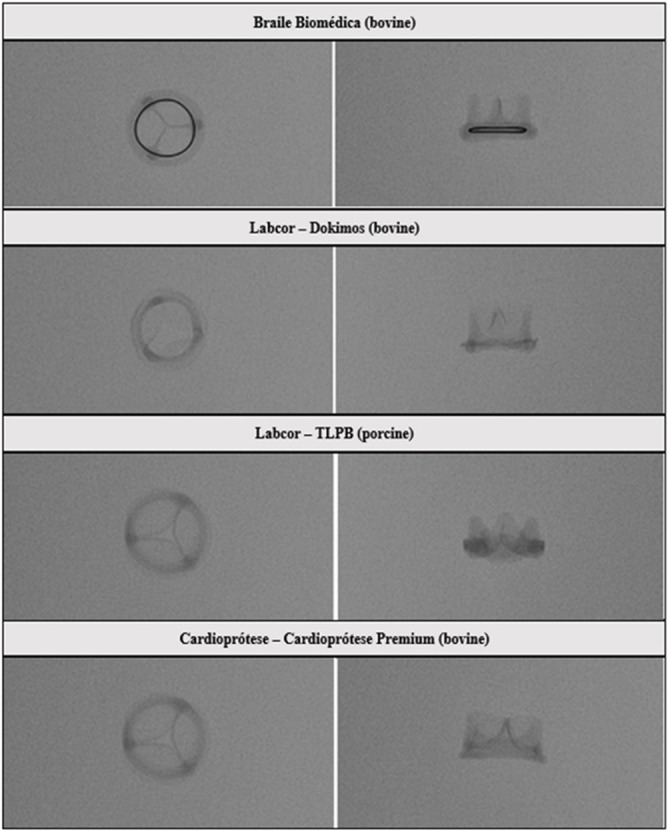
Radiography of the prostheses used in the test. Superior - inferior and lateral projections.

**Fig. 3:**
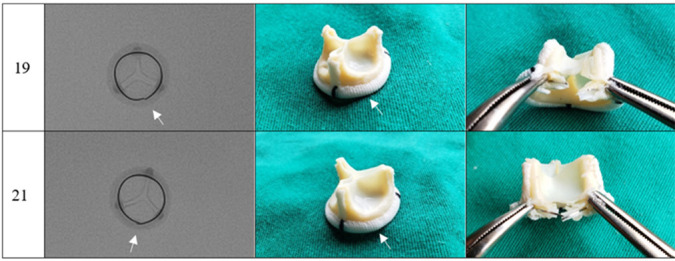
Braile prosthesis after fracture, sizes 19 mm and 21 mm. Radiography (superior-inferior projection) and photographs (intact lining and dissected lining). Arrow corresponding to the fracture site.

**Fig. 4:**
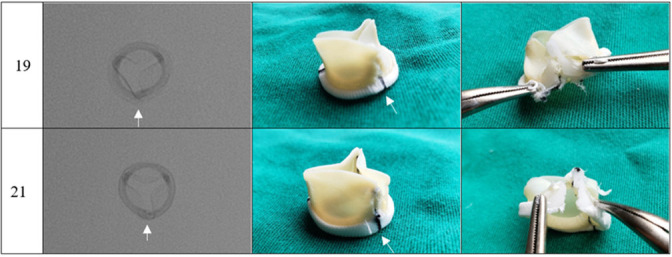
Labcor Dokimos prosthesis after fracture, sizes 19 mm and 21 mm. Radiography (superior-inferior projection) and photographs (intact lining and dissected lining). Arrow corresponding to the fracture site.

**Fig. 5:**
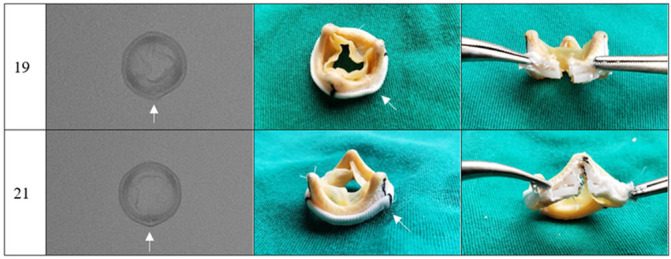
Labcor TLPB prosthesis after fracture, sizes 19 mm and 21mm Radiography (superior-inferior projection) and photographs (intact lining and dissected lining). Arrow corresponding to the fracture site.

**Fig. 6:**
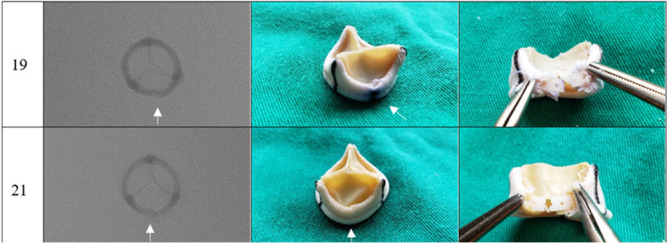
Cardioprótese Premium prosthesis after fracture, sizes 19mm and 21mm. Radiography (superior-inferior projection) and photographs (intact lining and dissected lining). Arrow corresponding to the fracture site.

The photographs and radiographs before the test allow the identification of the metallic ring in the models manufactured by Braile (Figures 1 and 2). After fracture, in all prostheses it was possible to evidence the site of the ring discontinuity, characterized by local deformity, which was more evident in the model with metallic ring (Figures 3 to 6).

A slight deformity in the ring structure was identified on palpation, keeping the Dacron reinforcement intact; no spicules were observed. After removing the Dacron lining and exposing the fracture site, linear discontinuity of the ring and absence of fragments were observed.

## DISCUSSION

In the present work it was evaluated - for the first time in an *ex vivo* test - the possibility of ring fracture of aortic bioprostheses manufactured in Brazil, when submitted to radial force by a high-pressure non-compliant balloon.

In VIV procedures, the use of strategies that reduce the postoperative transvalvular gradient aiming at values < 20 mmHg have an impact on symptom improvement, prosthesis durability, and mortality reduction^[5]^. The knowledge of the size of the dysfunctional prosthesis that receives the transcatheter prosthesis inside is part of the surgical planning, as well as the knowledge about the possibility of fracture in case of high postoperative gradient.

In several studies with *ex vivo* tests for ring fracture of bioprostheses^[9,11,12]^, it was observed that the presence of metallic reinforcement in some models of prostheses made them unbreakable (*e.g.*, St. Jude Trifecta and Medtronic Hancock II) or demanded a greater pressure for fracture. Because of this, in patients who need small-sized surgical prostheses (19 or 21 mm), if it is not possible to perform an aortic annulus enlargement technique, it should be considered to avoid the implantation of valves with metallic reinforcement, which may be difficult or impossible to fracture during VIV procedures.

### Limitations

Since the main objective of this study was to check the feasibility of bioprotheses ring fracture, its main limitations are the number of valves studied, with lack of valves with higher diameters (such as 23 mm), which were left out of this work because they generally do not course with severe mismatch (usually presented on the 19-21-mm valves). Ideally, further tests could be done with more valves for every size number of each different valve manufacturer, that would enable a statistical analysis which would give more credibility to the present findings. Besides that, the pressure values observed for a bench fracture may not correspond to the *in vivo* maneuver due to *in vivo* factors that may interfere with the prosthesis resistance, such as the mechanical stress accumulated over time that would reduce the resistance, or the scar tissue that could increase it. Interestingly, in a study that evaluated the fracture maneuver in 20 patients using Magna® model bioprostheses, the pressure needed to break it *in vivo* was on average 10 atm lower than the pressure in the bench study, while in the Mosaic® model the pressure needed for fracture *in vivo* was on average 5 atm higher than in the bench test^[7,9]^. Therefore, new studies must be addressed to determine the mean pressure required for the *in vivo* fracture, with different valve sizes and models.

Regarding the industry, the demonstration that the metal ring inside these valves makes their fracture more difficult creates the necessity of thinking about new solutions for reducing the breaking pressure in these procedures, especially in these smaller prostheses, thus increasing the possibilities of treating patients without other therapeutic options. Some recent models of bioprostheses have been developed to increase the orifice area in VIV implants by sliding a portion of its ring, thus allowing its remodeling, if necessary^[13]^.

The absence of projections of sharp materials or fragmentation of the polymeric framework of the prosthesis reduces the chances of complications resulting from the prosthesis rupture, which in the clinical application of the technique could lead to aortic root lesions or embolic phenomena.

In the experiments, the prostheses studied were photographed and radiographed. The images obtained (Figures 1 to 6) may serve as a reference to identify the model and assess the presence of metallic reinforcement, information of fundamental importance in planning VIV procedures.

## CONCLUSION

The present study revealed that the aortic bioprostheses produced by Brazilian companies, in a bench test, can be fractured using a non-compliant high-pressure balloon. However, this result does not allow the recommendation of the technique of ring fracture of bioprostheses in humans, and new studies must be done to warrant its security *in vivo*. The information obtained could assist in the planning of valve ring fracture in VIV procedures in patients with aortic valve bioprostheses.

**Table:** 

Authors’Roles & Responsibilities	
ALM	Substantial contributions to the conception or design of the work; or the acquisition, analysis or interpretation of data for the work; drafting the work or revising it critically for important intellectual content; agreement to be accountable for all aspects of the work in ensuring that issues related to the accuracy or integrity of any part of the work are appropriately investigated and resolved; final approval of the version to be published
DFG	Substantial contributions to the conception or design of the work; or the acquisition, analysis or interpretation of data for the work; drafting the work or revising it critically for important intellectual content; agreement to be accountable for all aspects of the work in ensuring that issues related to the accuracy or integrity of any part of the work are appropriately investigated and resolved; final approval of the version to be published
JHPF	Substantial contributions to the conception or design of the work; or the acquisition, analysis or interpretation of data for the work; drafting the work or revising it critically for important intellectual content; agreement to be accountable for all aspects of the work in ensuring that issues related to the accuracy or integrity of any part of the work are appropriately investigated and resolved; final approval of the version to be published
